# Supplementary material for: Routine metabolic rate is not associated with boldness in zebrafish
Source: Biol Open. 2026 Apr 7;15(3):bio062329. doi: 10.1242/bio.062329 (PMC13072135; doi:10.1242/bio.062329)
Supplement: Supplementary information [file biolopen-15-062329-s1.pdf]

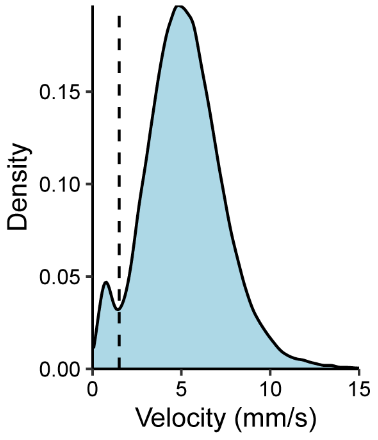


**Fig. S1.** Density plot of zebrafish velocity during exploration of a novel tank. Velocities below the dotted line were considered freezing/immobility.

**Table S1. Correlation between routine metabolic rate and behavioral parameters.**

| Strain | Sex | Behavior | Correlation | p-value | Statistic |
|---|---|---|---|---|---|
| TU | F | Bottom distance | -0.069 | 0.716 | Pearson |
| TU | F | Center distance | -0.093 | 0.623 | Spearman |
| TU | F | Distance travelled | 0.037 | 0.844 | Pearson |
| TU | F | Percent explored | 0.1 | 0.59 | Spearman |
| TU | F | Boldness index | 0.096 | 0.611 | Spearman |
| TU | F | Peak velocity | 0.19 | 0.313 | Pearson |
| TU | F | Freezing | 0.19 | 0.313 | Spearman |
| TU | M | Bottom distance | 0.12 | 0.526 | Spearman |
| TU | M | Center distance | 0.047 | 0.805 | Spearman |
| TU | M | Distance travelled | 0.22 | 0.249 | Spearman |
| TU | M | Percent explored | -0.056 | 0.768 | Spearman |
| TU | M | Boldness index | 0.0065 | 0.974 | Spearman |
| TU | M | Peak velocity | 0.22 | 0.241 | Spearman |
| TU | M | Freezing | -0.056 | 0.77 | Spearman |
| WIK | F | Bottom distance | -0.094 | 0.608 | Spearman |
| WIK | F | Center distance | 0.086 | 0.638 | Spearman |
| WIK | F | Distance travelled | 0.033 | 0.86 | Pearson |
| WIK | F | Percent explored | 0.015 | 0.934 | Spearman |
| WIK | F | Boldness index | -0.099 | 0.59 | Spearman |
| WIK | F | Peak velocity | -0.1 | 0.575 | Pearson |
| WIK | F | Freezing | 0.022 | 0.906 | Spearman |
| WIK | M | Bottom distance | 0.034 | 0.85 | Pearson |
| WIK | M | Center distance | 0.37 | 0.0357 | Spearman |
| WIK | M | Distance travelled | 0.049 | 0.788 | Pearson |
| WIK | M | Percent explored | -0.25 | 0.152 | Pearson |
| WIK | M | Boldness index | -0.16 | 0.385 | Pearson |
| WIK | M | Peak velocity | 0.12 | 0.49 | Pearson |
| WIK | M | Freezing | 0.25 | 0.158 | Spearman |

Biology Open • Supplementary information
